# Factors determining choice of place of delivery: analytical cross-sectional study of mothers in Akordet town, Eritrea

**DOI:** 10.1186/s12889-019-7253-8

**Published:** 2019-07-10

**Authors:** Nahom Kiros Gebregziabher, Almaz Yemane Zeray, Yordanos Tewelde Abtew, Tsinat Debesay Kinfe, Dawit Teweldemedhin Abrha

**Affiliations:** 1 School of Public Health, Asmara College of Health Sciences, Asmara, Eritrea; 2 Ministry of Health, Zoba Maekle Branch Office, Asmara, Eritrea; 3 Ministry of Health, Zoba Gash-Barka Branch Office, Barentu, Eritrea; 4 Department of Planning, Research and HRD Ministry of Health, Asmara, Eritrea

**Keywords:** Health facility delivery, Home delivery, Akordet, Eritrea

## Abstract

**Background:**

In Eritrea, facility delivery rates show great discrepancy within urban centers. This study was conducted in Akordet, a multi-cultural lowland town of Gash-Barka Region, aiming at assessing the factors influencing facility delivery.

**Methods:**

A community based analytical cross-sectional study was conducted among a total of 282 mothers who gave birth within the 2 years preceding the data collection time. Data collection was carried out by going house-to-house and interviewing the mothers using a structured closed ended questionnaire. Bivariate and multivariate logistic regressions were used to determine the magnitude of the relationship between place of delivery and the explanatory variables (Religion, Ethnicity, Mother’s educational level, Husband’s Educational level, Place of delivery preceding last pregnancy, Birth order of last child, Any complications during previous delivery, First ANC Visit during last pregnancy, Number of ANC visits during last pregnancy and Any complication during last pregnancy.). For this study, *p*-value ≤0.05 was considered as statistically significant.

**Results:**

The rate of facility delivery in this setting was found to be 82.3%. Almost all (96.1%) the mothers had at least one ANC visit during their last pregnancy, with the majority (59.7%) visiting ANC clinics during second trimester for the first time. Mothers whose educational level is junior and above (AOR 8.8, CI: 1.18–65.64), whose husband’s educational level is junior and above (AOR 3.92, CI: 1.03–14.54), who gave birth in health facility before the last pregnancy (AOR 8.16, CI: 3.41–19.48), and those who had complications during last pregnancy (AOR 2.24, CI: 1.04–4.82) were more likely to deliver in a health facility. Mothers whose last child’s birth order was 4th -6th were less likely (AOR 0.24, CI: 0.090.62) to deliver at health facility.

**Conclusions:**

Early initiation of ANC and regularity in attendance should be emphasized. Health educations given to pregnant mothers should try to persuade the mothers that each pregnancy and ensuing delivery is unique. Empowering the community in general and women in particular by increasing the level of participation in education might payoff in high level of facility delivery.

## Introduction

Maternal mortality has dropped by 45% worldwide, from 1990 through 2013. [1] However, globally about 830 women die every day due to preventable pregnancy related causes [2], 99% of this occurring in developing countries. [3] The maternal mortality ratios (MMR) show huge discrepancies between the developed (16 per 100,000 live births) and the developing nations (240 per 100,000 live births), and more than half (56%) of these maternal deaths occur in the sub-Saharan Africa. [4] Furthermore, the maternal mortality shows grater discrepancy within sub-Saharan countries. [5, 6] The large number of maternal deaths, especially in developing countries, has been attributed to the low levels of maternal health care seeking behavior, as evidenced by low proportions of antenatal care utilization and extremely low deliveries attended by a skilled-person. [7, 8]

The quality of child delivery outcome can only be enhanced through proper antenatal-care (ANC) coupled with better choice of place of delivery as recommended by health authorities. [9–11] Furthermore, place of delivery is found to be one of the key predictors of neonatal mortality. [12] The World Health Organization (WHO) has been recommending at least four antenatal care (ANC) visits during pregnancy, and that postnatal care should be provided at 6 h, 6 days, 6 weeks, and 6 months after childbirth to ensure women’s physical and mental wellbeing. [13] As such, chances are very low for the women who gave birth at home to get this important postnatal care. [14]

Despite the encouraging trends in antenatal care service utilization coverage, delivery in health facilities is still challenging in developing countries. Several studies have presented that poor availability of resources and services as the major cause of underutilization of maternal health services. [15, 16] Nevertheless, in some settings, even if the services are readily available, these facilities are not always available to women of some socio-economic classes. [17]

Access to and utilization of available skilled birth attendance services is hampered by a number of factors including social and cultural contexts, religious beliefs, inadequate skilled health personnel, and long distances to health facilities. [1, 6] This means, there is still a lot of work that needs to be done in order to register significant increase in skilled birth attendance.

Eritrea is one of the three Sub Saharan African countries that has achieved the Millennium Development Goal 5 [18] and has made significant progress in maternal health by reducing the maternal mortality (MMR) from 998/100,000 in 1995 [19] to 352/100,000 in 2010 [20]. However, the current average annual decline rate of 6.5%, 1990–2015, is projected to ask Eritrea, two decades to match with the global average MMR of 210/100,000 [18]. This requires the country to invest a lot of resources into the Maternal and Child Health program to reach the Sustainable Development Goal 3, which calls reduction of global maternal mortality ratio to less than 70 per 100,000 live births and to end preventable deaths of newborns and under-five children by the year 2030. [21] A study done in Eritrea, back in 2004, reported that 16% of maternal deaths occurred during pregnancy, 48% during childbirth, and 36% during the postpartum period. This study also identified four delays as the causes of the maternal death; failure or delay in recognition of danger signs, delay in deciding to seek care, delay in reaching appropriate care, and delay in receiving appropriate care. [22]

Although antenatal care coverage in Eritrea is very high, ranging from 84.2% in rural areas to 97.3% in urban areas, skilled birth attendance in health facilities is still very low, from 16.5% in rural to 73.2% in urban settings. [20] This low level of health facility delivery rate is occurring despite the fact that roughly each community in Eritrea now has maternal and child health (MCH) services free of charge. All urban centers have at least one health center within a reasonable distance for the majority of the residents, but still the 2010 Eritrean Population and Health Survey (EPHS) shows a great discrepancy in the rate of facility delivery within urban centers, from 92.5% in the capital, Asmara, to an average of 62.5% in other towns. [20] This suggests that factors other than availability and accessibility of health facilities are playing in the background to reduce facility delivery attendance. A qualitative study conducted by Chol et al. [23] in Asmara, Eritrea, recognized two main factors as facilitators of women’s access and utilization of maternal health services. These factors were health education, either through mass media or sessions in the health facility, and women’s empowerment. Another study conducted in rural Eritrea identified several socioeconomic and demographic factors, related both to the mother and the husband, as determinants of facility delivery. In addition, this study identified some health facility related factors, like distance and quality of care, to affect choice of place of delivery. [24] This calls for localized evidence on determinants of place of delivery to understand the phenomenon. This is vital in identifying key priority areas for policy and practice, and increasing the rate of facility delivery.

Gash-Barka, the largest administrative region in the country, is where the lowest rate of facility delivery was observed, with only 18% of women giving birth in health facilities, and the most common reason mentioned being distance to health facility. [20] This study was conducted in Akordet, a multi-cultural lowland town of Gash-Barka region, aiming at assessing the socio-demographic factors of women which influence facility delivery.

## Data and methods

### Study design and setting

A community based analytical cross-sectional study was conducted on December 2017 in the town of Akordet, Eritrea. The town has one hospital and one health center that provides maternal and child health services. The target population was women who have had at least one delivery. Women who gave birth within 2 years preceding the data collection date and who were permanent residents of the town were included in this study. Severely sick, mentally ill and primigravida women, who were pregnant during the data collection time, were excluded from the study.

### Sample size and sampling technique

Initially, this study was meant to be a census type of study. According to the town Administration Office, there were a total of 313 women who gave birth within the 2 years preceding the data collection time. Based on the data from the Office, a list of eligible mothers was prepared. Once the study participants were identified, community health agents helped data collectors to identify individual mother’s house. However, in this study, a total of 282 mothers, who fulfilled the inclusion criteria, were included. The main reason for the failure of women from participating in the study was unavailability of the mother at home during data collection time.

### Data collection tools and methods

Data collection was carried out by interviewing the mothers using a structured closed ended questionnaire. The questionnaire was developed after a vigorous review of literature on determinants of place of delivery in similar settings. This questionnaire was composed of several parts, which included socio-demographic characteristics of the mothers, ANC visits, pregnancy and child birth experience, and place of delivery. The questionnaire was distributed to experts on the field for content assessment in respect to the study setting. Then pretest of the questionnaire was conducted 2 weeks prior to the actual data collection time, to make the data collection tool more understandable and culture sensitive. Based on the insight gained during the pretest the question “income of the household”, which is sensitive, was changed with “perceived wealth index”. The reliability of the questionnaire was identified using Guttman’s λ_3_ coefficient, and it was found to be 0.76.

### Outcome and explanatory variables

The mothers were asked about the place of their last delivery, which had a dichotomous outcome, either home or health facility. Thus, in this study place of delivery was the outcome variable. Based on a review of the literature and the study setting, we selected to analyze 17 explanatory variables which have the potential to influence place of delivery. This includes; maternal age, marital status, sex of household head, religion, ethnicity, maternal education, husband education, perceived wealth class, ownership of means of transportation, birth order of last child, adverse birth outcomes during delivery preceding the last pregnancy, regular ANC visit during the last pregnancy, time of the first ANC visit, number of total ANC visits during the last pregnancy, any pregnancy related problem during the last pregnancy, and sex of ANC provider.

### Data analysis method

After checking the data for completeness, it was coded and feed into Statistical Package for Social Sciences version 23 for analysis. Simple frequencies and proportions were used to describe the socio-demographic and pregnancy related characteristics of the respondents and Chi-square test was used to assess the relationship between place of delivery and the explanatory variables. Variables found to have statistically significant association with the dependent variable, during chi-square analysis, were further assessed by using bivariate and multivariate logistic regression to determine the magnitude of the relationship between the outcome variable and the independent variables, if there was any. For this study, *p*-value ≤0.05 was considered as statistically significant.

## Results

This study included a total of 282 mothers. The age of the participants ranged between 17 and 42 years, with a median age of 27 and inter-quartile range of 8 [IQR 23–31]. Women who were in the age group of 26 up to 35 years accounted the highest proportion (47.5%) of the participants. Almost all the mothers were married (96.5%), and male headed households accounted 66.7%. Most (78.7%) of the participants were Muslims and from the Tigre ethnic group (68.1%). Almost half (49.6%) of the mothers had elementary level of education and 91.8% were housewives. Ownership of Means of Transportation was found to be only 8.5% and most (64.2%) of the participants perceived their wealth class to be “Middle Class”. Most of the husbands’ had educational level of elementary and above, and 42.6% were government employees. (Table 1).

**Table 1:** Descriptive data on the study population (*N* = 282)

Variable	Category	Frequency	Percentage
Age Group	17–19	15	5.3
	20–25	103	36.5
	26–35	134	47.5
	> = 36	30	10.6
Marital Status	Single	7	2.5
	Married	272	96.5
	Divorced	3	1.1
Sex of Household Head	Male	188	66.7
	Female	94	33.3
Religion	Moslem	222	78.7
	Christians	60	21.3
Ethnicity	Tigrinya	68	24.1
	Tigre	192	68.1
	Bilen	15	5.3
	Kunama	3	1.1
	Saho	4	1.4
Mother Educational Level	Illiterate	74	26.2
	Can Read and Write	16	5.7
	Elementary	140	49.6
	Junior And Above	52	18.4
Mother Occupation	Housewife	259	91.8
	Daily Worker	4	1.4
	Merchant	4	1.4
	Government/ Private Employee	15	5.3
Husband Educational Level	Illiterate	66	23.4
	Can Read and Write	38	13.5
	Elementary	91	32.3
	Junior and Above	87	30.9
Husband Occupation	Farmer	60	21.3
	Daily Worker	83	29.4
	Private Employee	19	6.7
	Government Employee	120	42.6
Ownership of Means of Transportation	Yes	24	8.5
	No	258	91.5
Perceived wealth class	Poor	87	30.9
	Middle Class	181	64.2
	Rich	14	5.0

The most common (68.8%) age at first delivery was between 18 and 24 years old, with only 7.4% of the mothers giving their first birth after they reached their 25th birthday. However, 34.4% of the mothers were 30 years old or above, and 7.4% were between the ages of 16–19 years old when they gave their last birth. Majority (57.8%) of the mothers’ last child’s birth order was 1st to 3rd and 12.8% of the mothers had complications during delivery preceding the last pregnancy. Only 35.5% of the mothers gave birth in health facility preceding the last delivery. However, during the last delivery this number rose to 82.3%. The most common reasons mentioned for home delivery were lack of Transportation and the Comfort to give birth at home, 46 and 32% respectively. Out of all the participants only 1.1% received postnatal care. The mothers were asked as to the “ideal place of delivery” and 64.9% said at health facility, 29.1% said at home by skilled birth attendant while 6% opted for home delivery by a traditional birth attendant (TBA). (Table 2).

**Table 2:** Descriptive data on delivery related domains of the study group (*N* = 282)

Variable	Category	Frequency	Percentage
Age at first delivery	12–17	67	23.8
	18–24	194	68.8
	> = 25	21	7.4
Age at last delivery	16–19	21	7.4
	20–29	164	58.2
	> = 30	97	34.4
Birth order of last child	1–3	163	57.8
	4–6	92	32.6
	> = 7	27	9.6
Any complication during delivery preceding last pregnancy	Yes	36	12.8
	No	246	87.2
Place of delivery preceding the last pregnancy	Health facility	100	35.5
	Home	182	64.5
Place of Last Delivery	Home	50	17.7
	Health facility	232	82.3
Reason for home delivery (*n* = 50)	Labor was easy	2	4.0
	Comfort	16	32
	Lack of Transport	23	46
	Shyness	3	6.0
	Family refusal	3	6.0
	I don’t know the importance	3	6.0
Received Postnatal Visit	Yes	3	1.1
	No	279	98.9
Ideal place for delivery	Home by TBA	17	6.0
	Home by Health professional	82	29.1
	Health facility	183	64.9

Almost all (96.1%) of the mothers had at least one ANC visit during their last pregnancy, with the majority (59.7%) of them visiting ANC clinics during the second trimester of the pregnancy for the first time. Of these mothers, only 70% reported to be regular attendees of ANC services and only 58.1% had visited ANC clinics for four times or more. Majority (89.7%) of the participant were attended in ANC clinics by male health workers. The most common reasons mentioned for not attending ANC were “lack of knowledge” (26.7%) and being “busy at home” (26.7%). (Table 3).

**Table 3:** Descriptive data on ANC related domains of the study group during last pregnancy

Variable	Category	Frequency	Percentage
ANC visit (n = 282)	Yes	271	96.1
	No	11	3.9
Trimester during first ANC visit (*n* = 273)	First	56	20.5
	Second	163	59.7
	Third	54	19.8
Regularly visit ANC service (n = 273)	Yes	82	70.0
	No	191	30.0
Number of ANC visits (n = 273)	1–3	114	41.9
	4–6	128	47.1
	> = 7	31	11.00
Reasons for not visiting ANC services (*n* = 9)	Lack of Knowledge	4	26.7
	Transportation problem	2	13.3
	Family refusal	2	13.3
	Did not feel bad	1	6.7
	Busy at home	4	26.7
	Feel shy	2	13.3
Sex of the ANC provider	Male	245	89.7
	Female	28	10.3

### Bivariate analysis

In this study seventeen explanatory variables were set to be assessed, out of which eleven were found to be significant predictors of place of delivery using bivariate analysis (*p ≤ 0.05*). Mothers whose religion is Christian (COR 2.79, CI: 1.05–7.39), had elementary level of education (COR 2.24, CI: 1.13–4.45) or junior and above (COR 9.91, CI: 2.2–44.4) level of education, have husband with junior and above level of education (COR 5.45, CI: 2.03–14.62), who gave birth in health facility before the last pregnancy (COR 3.85, CI:2.03–7.28), who had experienced complications during previous deliveries (COR 2.22, CI: 1.06–4.68), who had visited ANC for 4 to 6 times (COR 2.33, CI:1.2–4.5) or ≥ 7 times (COR 5, CI:1.12–22.27), and who had complications during last pregnancy (COR 1.9, CI:1.02–3.52) were more likely to deliver at health facility. On the other hand, mothers from Tigre (COR 0.34, CI: 0.12–0.91) or the other ethnicities (COR 0.11, CI: 0.03–0.39) were less likely to deliver in a health facility as compared to the mothers from the Tigrinya ethnic group. In addition, mothers whose last child’s birth order was 4th- 6th (COR 0.37, CI: 0.18–0.73) or ≥ 7th (COR 0.24, CI: 0.09–0.63), and who made their first ANC visit, during their last pregnancy, at some stage in the third trimester (COR 0.18, CI: 0.05–0.59) were less likely deliver at health facility. (Table 4).

**Table 4:** Regression results for determinants of health facility delivery among women in Akordet Town

Exposure variable	Variables	Unadjusted Odds Ratio (COR) [95% CI]	Adjusted Odds Ratio (AOR) [95% CI]
Religion	Moslem	1	1
	Christian	2.79 [1.05–7.39]*	0.55 [.03–8.68]
Ethnicity	Tigrigna	1	1
	Tigre	0.344 [0.12–0.91]*	0.78 [.05–11.38]
	Other	0.11 [0.03–0.39]**	0.11 [0.01–1.73]
Mother educational level	Illiterate	1	1
	Can read and write	0.66 [0.21–2.04]	0.99 [0.27–3.66]
	Elementary level	2.24 [1.13–4.45]*	1.52 [0.59–3.95]
	Junior and above	9.91 [2.2–44.4]**	8.80 [1.18–65.64]*
Husband Educational level	Illiterate	1	1
	Can read and write	1.3 [0.52–3.26]	0.93 [0.3–2.9]
	Elementary level	1.89 [0.88–4.04]	1.59 [0.55–4.53]
	Junior and above	5.45 [2.03–14.62]**	3.92 [1.06–14.54]*
Place of delivery preceding last pregnancy	Home	1	1
	Health Facility	3.85 [2.03–7.28]***	8.16 [3.41–19.48]***
Birth order of last child	1st -3rd	1	1
	4th -6th	0.37 [0.18–0.73]**	0.24 [0.09–0.62]**
	7th and above	0.24 [0.09–0.63]**	0.032 [0.1–1.04]
Any complications during pervious delivery	No	1	1
	Yes	2.22 [1.06–4.68]*	0.64 [0.21–1.98]
First ANC visit during last pregnancy	First Trimester	1	1
	Second Trimester	0.35 [0.11–1.06]	0.46 [0.09–2.2]
	Third Trimester	0.18 [0.05–0.59]**	0.038 [0.06–2.35]
Number of ANC visits during last pregnancy	1–3	1	1
	4–6	2.33 [1.2–4.5]*	2.06 [0.8–5.3]
	7 and above	5 [1.12–22.27]*	2.41 [0.28–20.52]
Any complication during last pregnancy	No	1	1
	Yes	1.9 [1.02–3.52]*	2.24 [1.04–4.82]*

* P ≤ 0.05, ** P ≤ 0.01, *** P ≤ 0.001

### Multivariate analysis

Out of the eleven variables which were found to have a statistically significant association with place of delivery, in the bivariate analysis, only five variables remained to be significant predictors of place of delivery in the multivariate analysis. Mothers whose educational level is junior and above (AOR 8.8, CI: 1.18–65.64), whose husband’s educational level is junior and above (AOR 3.92, CI: 1.03–14.54), who gave birth in health facility before the last pregnancy (AOR 8.16, CI: 3.41–19.48), and those who had complications during last pregnancy (AOR 2.24, CI: 1.04–4.82) were more likely to deliver in a health facility as compared to their counterparts. Those mothers whose last child’s birth order was 4th -6th were less likely (AOR 0.24, CI: 0.090.62) to deliver at health facility. (Table 4).

## Discussion

This study found that the proportion of mothers who gave birth at a health facility to be 82.3% in this urban setting. This proportion is much higher than the zonal and overall urban average of 18 and 63% respectively and slightly lower than that of Asmara, the capital city, which was 93% in 2010. [20] The reason for this high proportion of facility delivery could be due to increased awareness of the population since 2010, given the fact that the proportion of facility delivery within this study group increased from 35.5% in previous delivery to 82.3% during the last delivery. Another reason could be due to the fact that this study included only women living in the town of Akordet, who can have relatively good access to health facilities and transportation services as compared to women living in the satellite settlements of the sub-zone. This high facility delivery rate in this setting, which approximated the rate in Asmara, is commendable, as, though classified as urban centers, the two settings markedly defer in their level of urbanization. The rate of facility delivery in this study was higher when compared to study findings from other similar settings, like local studies from Ethiopia (73.2%), Nigeria (65%), Tanzania (56%) and Kenya (42%). [25–27] This could be due to the inherent difference between these setting in accessibility to health facility and socio-cultural structure.

Transportation problem was the most frequently mentioned reason by the mothers who gave birth at home. This seems to be a legitimate problem as 91.5% of the mothers in this study reported to have no means of transportation of their own. This is supported by the 2010 EPHS finding where distance was mentioned as a barrier for facility delivery by 40% of Gash-Barka women. [20] The second most common reason for home delivery was “comfort”, mentioned by 32% for the mothers, which seems an opinion of most of the mothers in this study; as almost third of them mentioned home delivery with skilled birth attendant as an “ideal” environment for childbirth. These women who chose home delivery might have had previous experience with home delivery that had no complications, and therefore, opted for the same place, where they can be comforted in a familiar setting with their beloved ones neighboring them during delivery. [28] In this study home delivery by TBAs was mentioned as an ideal setting for child delivery by 6 % of the participants. Taking into account the study setting and the high ANC coverage, the reason for this choice can be attributed to the prevailing faith in the skills and expertise of TBAs, easy availability when needed and the traditional views and religious beliefs regarding delivery as in many other settings. [29–31]

The ANC service attendance seems to be a common place in this setting as 96.1% of the study participants had at least one visit. Again this finding is higher than that of the zonal average of 84.7%, the national average of 89% [20] and the Sub-Saharan Africa average of (49%) [8]. However, the regularity in attendance (70.0%) and the proportion of mothers having the recommended four or more ANC visits, which was 58.1%, though higher than the national average of 57.4%, is not satisfactory as compared to the 74.9% for women living in other towns of the country. [20] The reason for this reduction could be the way women perceive the whole concept of ANC, aside from getting some material support like bed-nets which are distributed at first contact with the pregnant mother. Another factor can be the timing of first ANC visit, the later the mother’s first visit the lower the chance for her to have four or more visits. This is supported by other finding from this study, as only 20.5% of the mothers had their first ANC visit during the first trimester. This proportion was lower than the national and urban-setting average of 26.6 and 39.1% respectively [20] and 25.5% of an Ethiopian study [32]. Several reasons have been put forward as to why pregnant women start ANC later during pregnancy, most of them being related to socioeconomic factors. [33] However, this needs to be studied further in this setting. This low frequency and irregularity of ANC visit needs to be dealt with as antenatal care remains to be a vital health care tool to reduce the risk of pregnancy and delivery related complications. [34, 35] The most common reasons for not attending ANC in this study were being busy with home chores and lack of knowledge on ANC, mentioned by 26.7% of the participants who never attended ANC.

### Factors associated with facility delivery

Several socio-demographic and personal experiences related factors were found to determine the place of delivery on logistic regression analysis. Religion was one of the factors influencing institutional delivery in this setting. Mothers who are Christians were 2.7 (COR) times more likely to deliver in health facility. This was similar with studies done in Nigeria and Ethiopia where Christian mothers were more likely to deliver in a health facility as compared to Muslim mothers. [36–38] On the other hand, a qualitative study conducted to uncover the effect of religion on place of deliver found no difference in preference between Muslim and Christian mothers. [39] This calls for a better-quality analysis of the pathways in which religion affects the choice of place of delivery. Another factor affecting place of delivery was mother’s negative experience in previous deliveries, as women with this experience were found to have 2.2 (COR) times higher odds of delivering in health facility. This indicates that women’s personal belief, as to the safety of oneself during delivery, can be the main reasons for why women choose to get facility or skilled delivery. [40] In this study only 64.9% of the mothers mentioned health facility as the “ideal place” to give birth, despite the large proportion of facility delivery. This indicates that facility delivery could be high, even if the clients prefer to deliver at home, because of safety reasons.

Educational level is also one of the most important factors in determining selection of place of delivery. Women with educational level of junior and above were 8.8 (AOR) times more likely to give birth in health facility as compared to those who have lower levels of education. Similarly, husbands’ educational level was also important in determining place of delivery as women whose husbands had junior and above level of education found to have higher odds of facility delivery (AOR = 3.92). This finding was similar with results of several studies where educational level of the mother and her partner was found to significantly affect the choice of place of delivery. [37, 41–44] The reason for this could be the effect of educational level in increasing the person’s cognitive ability to comprehend health education materials which in turn helps the persons to take better health choices, though the effects might be direct or indirect. [45, 46]

Increased frequency of ANC visit was associated with facility delivery as mothers who had four or more ANC visits were 2.7 (COR) times more likely to deliver at health facility as compared to those who had less than four visits, which is consistent with other study findings. [47, 48] This could be due to the fact that increased contact time with health care providers creates a platform to put emphasis on the importance of facility delivery [34, 49] or because those mothers who have high ANC visits are those who already have good health seeking behavior. These women with high ANC visit could also be mothers with some pregnancy related complications, who were told to visit ANC frequently by the health care providers and subsequently told to delivery at a health facility. This can be supported by the fact that, in this study women who had any pregnancy related complication were found to be 1.9 (COR) times more likely to delivery at health facility. Nonetheless, the relationship between increased number of ANC visit and facility delivery implies the WHO’s new guideline, which requires every pregnant mother to have at least 8 ANC visits, [50] will contribute towards increased facility delivery.

Previous experience of facility delivery was found to be a strong predictor of place of delivery, as those women who delivered in health facility during the pregnancy preceding the last one were 8.6 (AOR) times more likely to choose the same setting for the next delivery. This could be influenced by being familiar with the heath facility delivery setting and the trust the mothers built on it. Birth order of the last child was also one of the variables which predict place of delivery. As the birth order of the child increases, the probability of facility delivery decreases. As compared to the mothers whose child’s birth order was 1st to 3rd, those with 4th to 6th were 76% (AOR 0.24) less likely to give birth in a health facility. This was similar with the EPHS 2010 [20] findings, where Forty-eight percent of first order births delivered in health facilities, as compared to only 23% of the 6+ birth orders, and findings from other studies. [43, 51]

Another factor that was found to negatively affect the probability of facility delivery was the trimester in which the mothers visited ANC for the first time. Mothers who visited ANC during the last trimester were 0.18 times less likely to deliver at hospital. This finding was similar to a study conduced in rural Ghana, where it was found women who stared ANC visit after the second trimester were less likely to have facility delivery. [48] This seems like mothers who go to ANC during the end of the pregnancy are doing so, to check their status before deciding the place of delivery, as it was found in a phenomenological study of home delivery conducted in Ethiopia. [52]

The effect of ethnicity in determining the place of delivery in this setting was assessed and the results of bivariate logistic regression showed that mother from the Tigre and other ethnic groups were 65.6 and 89% less likely to give birth in a health facility as compared to the mothers from the Tigrinya ethnic group. This difference could be attributed to the fact that the people of the Tigrinya ethnic group in this study setting are either a government workers or engage in business, which increases their probability to be affluent. Affluence was found to be one of the most important determinants of facility delivery in many developing countries. [15, 20, 53] Furthermore, since these ethnicities differ culturally and religion wise, Tigrinya mothers, who came from other place, may not easily get TBAs who knows their tradition of delivery. This may force them to opt to use facility delivery, although it requires further research to understand the direct effect of culture and religion on choice of place of delivery in this setting.

### Limitation of the study

The study was conducted among women living within the town of Akordet, which limits its ability to generalize about women living in the Sub-Zone. There might be some recall bias with regard to initiation and frequency of ANC service. In addition, this study was not able to assess the wealth quintile of the family objectively, rather used perceived wealth class, which may affect the result of the statistical test.

## Conclusion

This study founds out that the rate of facility delivery in Akordet town to be high. The ANC coverage was universal with limitations in frequency and regularity of checkups. Emphasis should be put on the importance of starting ANC visits as early as possible. Mother’s level of education, husband’s level of education, place of preceding delivery, birth order of last child and complications during pregnancy were independent predictors of place of delivery in the study setting. The choice of home delivery with health professional was high in this community which needs to be given due attention, since comfort was one of the main reasons for home delivery. The issue of transportation during the time of delivery, which was the main reason for home delivery, should also be addressed by providing ambulance services to help the mothers get easy physical access to health facilities. To address the effect of religion and culture on utilization of maternal health services, health education programs on maternal health should include religious leaders and community elders as target audiences. Health educations given to pregnant mothers, through mass media and in ANC clinics, should persuade the mothers that each pregnancy and the ensuing delivery is unique, in order to avert deterioration on personal appraisal of the need for facility delivery in subsequent births. Empowering the community in general and women in particular by increasing their level of participation in education might payoff in high level of facility delivery. The findings of this study call for a prompt adoption and enforcement of the new WHO guideline on ANC by the health authorities, at all levels of health services delivery. In addition, it is empirical to study the need and the feasibility of introducing skilled-person assisted home delivery, by learning from experiences of settings that have already adopted it. Finally, national public health policies should give room for local and context specific community requirements to make the maternal health services acceptable to local consumers.

## Data Availability

The data set analyzed during this study is available from the corresponding author on reasonable request.

## Abbreviations

ANC: Antenatal Care
AOR: Adjusted Odds Ratio
COR: Crude Odds Ratio
EPHS: Eritrean Population and Health Survey
MCH: Maternal and Child Heath
MMR: Maternalmortality ratios
TBA: Traditional Birth Attendants
WHO: World Health Organization
